# Variation in Host and Pathogen in the *Neonectria/Malus* Interaction; toward an Understanding of the Genetic Basis of Resistance to European Canker

**DOI:** 10.3389/fpls.2016.01365

**Published:** 2016-09-15

**Authors:** Antonio Gómez-Cortecero, Robert J. Saville, Reiny W. A. Scheper, Joanna K. Bowen, Hugo Agripino De Medeiros, Jennifer Kingsnorth, Xiangming Xu, Richard J. Harrison

**Affiliations:** ^1^NIAB-EMRKent, UK; ^2^School of Agriculture Policy and Development, University of ReadingReading, UK; ^3^The New Zealand Institute for Plant and Food Research LimitedHavelock North, New Zealand; ^4^The New Zealand Institute for Plant and Food Research LimitedAuckland, New Zealand; ^5^Empresa de Pesquisa Agropecuária e Extensão Rural de Santa CatarinaCaçador, Brazil

**Keywords:** European canker, pathogenicity test, *Neonectria ditissima*, phylogenetics, disease resistance

## Abstract

Apple canker caused by the phytopathogenic fungus *Neonectria ditissima* is an economically important disease, which has spread in recent years to almost all pome-producing regions of the world. *N. ditissima* is able to cross-infect a wide range of apple varieties and causes branch and trunk lesions, known as cankers. Most modern apple varieties are susceptible and in extreme cases suffer from high mortality (up to 50%) in the early phase of orchard establishment. There is no known race structure of the pathogen and the global level of genetic diversity of the pathogen population is unknown. Resistance breeding is underway in many global breeding programmes, but nevertheless, a total resistance to canker has not yet been demonstrated. Here we present preliminary data from a survey of the phylogenetic relationships between global isolates of *N. ditissima* which reveals only slight evidence for population structure. In addition we report the results of *four* rapid screening tests to assess the response to *N. ditissima* in different apple scion and rootstock varieties, which reveals abundant variation in resistance responses in both cultivar and rootstock material. Further seedling tests show that the segregation patterns of resistance and susceptibility vary widely between crosses. We discuss inconsistencies in test performance with field observations and discuss future research opportunities in this area.

## Introduction

European canker (caused by *Neonectria ditissima)* is one of the most destructive diseases of apple and pear. The fungus attacks trees in the orchard, causing cankers and dieback of young shoots, resulting in loss of fruiting wood and increased pruning costs (Swinburne, 1975). Apple canker can be particularly damaging in young orchards where, in some years, up to 10% of trees can be lost annually in the first few years of orchard establishment as a result of trunk cankers (Angela Berrie, personal communication). In some regions of the world (i.e., Northern Europe) *N. ditissima* can also cause a fruit rot in stored fruit. The rot, which is often found at the fruit stalk end, is difficult to spot on the grading line, but becomes obvious during marketing leading to rejection of fruit consignments (Xu and Robinson, 2010).

The taxonomic history of the pathogen is somewhat complex, having altered in name repeatedly over the past 150 years. Studies based largely on host range and morphology have at various times divided the original pathogen (named *Neonectria ditissima* Tul. and C. Tul, Tulasne and Tulasne, 1865) into two separate species, *Nectria ditissima* and *Nectria galligena* (Bres.) (Cayley, 1921) and later renamed *Neonectria galligena* (Bres.) before returning to its original name some 10 years ago (Castlebury et al., 2006). The anamorphic state is *Cylindrocarpon heteronema* (Berk. and Broome) Wollenw. 1916.

The host range of *N. ditissima* encompasses multiple hardwood trees species such as *Fagus, Populus, Acer, Salix*, and *Betula* species (Castlebury et al., 2006; Walter et al., 2015). Phylogenetic studies have revealed that European and American populations appear to have a significant level of nucleotide divergence at ß-tubulin and RPB2 loci (Castlebury et al., 2006), indicating that the populations may be allopatrically isolated. American populations of *N. distissima* have been shown to contain abundant within-population diversity (Plante et al., 2002), which led to the hypothesis that America is the center of origin of *N. ditissima*. However, as stated by Castlebury, without further sampling in Europe (despite recent work) this cannot yet be confirmed (Ghasemkhani et al., 2016).

**Figure 1:**
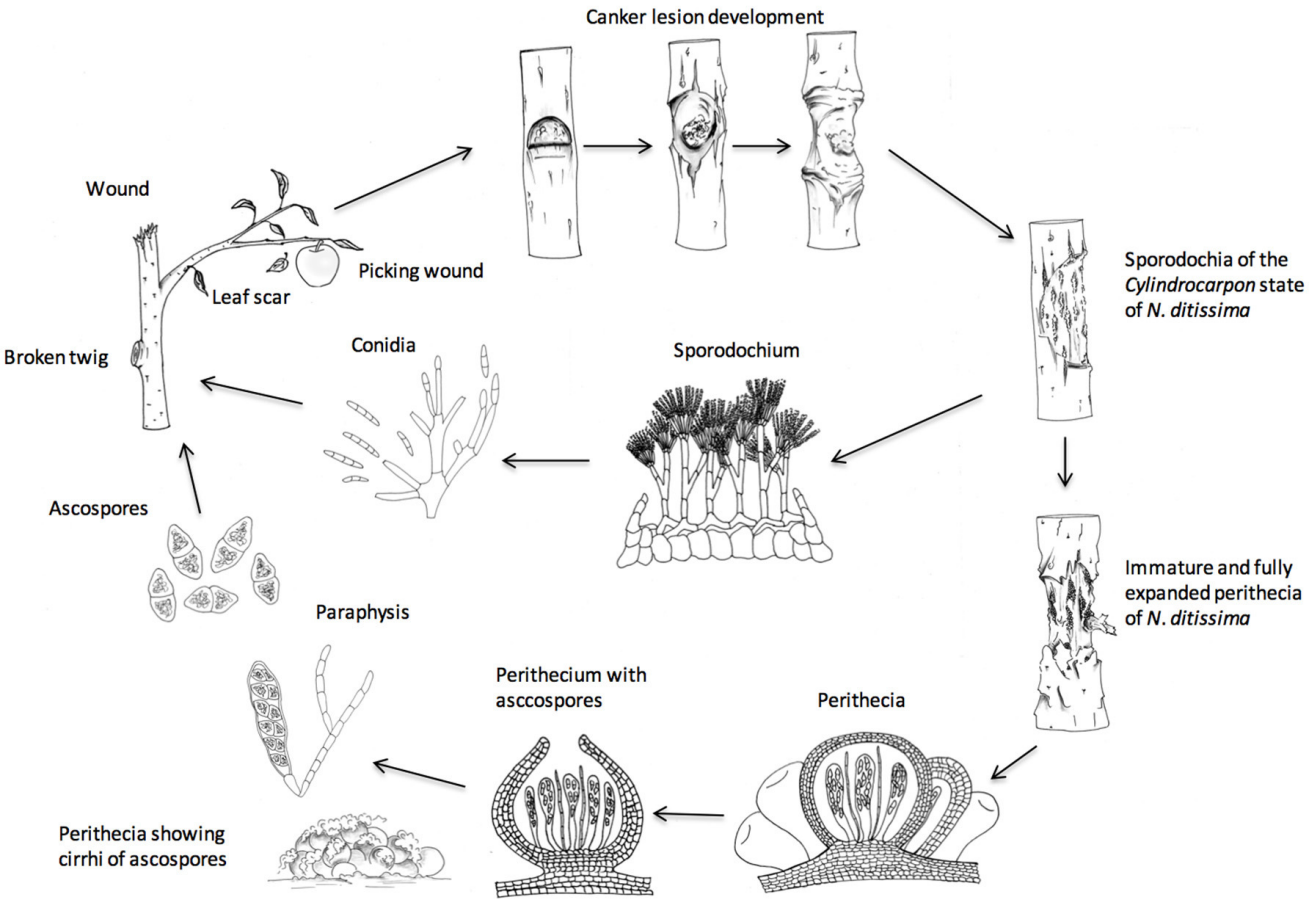
**Life cycle of ***Neonectria ditissima***- after Agrios 1997**.

Much is known about the epidemiology of the disease in the orchard (see Figure 1 for a graphical depiction of the lifecycle). The fungus produces two spore types, conidia (imperfect/asexual spores) and ascospores (sexual/perfect spores). Conidia are generally produced within the first year of canker formation when the temperature increases in the spring and summer and are spread throughout the season by rain splash. By contrast, ascospores are mainly produced by old canker lesions during the autumn, winter and spring and are discharged during rain, and wind- or splash-dispersed. Both spore types enter through wounds, either natural such as bud-scale scars, leaf scars, fruit scars or artificial such as pruning wounds. Thus, inoculum and points of entry on the tree are available all year round (Amponsah et al., 2015) and the only limiting factor is rain, which is essential for spore production, spread, germination and infection (Xu et al., 1998). The disease is most destructive in young trees infected with canker, as latent infections appear as systemic infection and trunk cankers several years after planting (McCracken et al., 2003). Factors that affect canker expression are not understood but possibly relate to stress (cold, drought, water-logging and herbicide applications), or fertilizer applications. For example, post-harvest foliar nitrogen applications increased leaf scar infections six- to nine-fold in New Zealand orchards (Dryden et al., 2016). New cultivars being planted in the UK such as “Scifresh,” “Cameo,” “Kanzi,” “Zari,” “Rubens,” and older cultivars such as “Gala” and “Braeburn” are all very susceptible to *N. ditissima* and the development of systemic canker in young orchards leading to tree loss is a significant problem, with severe financial loss particularly in modern intensive planting systems (Weber, 2014). In contrast to the orchard, the epidemiology of *N. ditissima* in the nursery is not understood and infected trees are rarely seen in nursery production so it is assumed that the disease is present as a latent infection (McCracken et al., 2003).

Currently canker is controlled in the orchard by a combination of cultural methods to remove canker lesions and the use of protectant fungicides. However, Cooke showed that even the most stringent fungicide programmes only reduce the increase of canker incidence but canker incidence continued to increase (Cooke, 1999). Therefore, this approach does not seem to prevent the fungus from invading the trees, causing cankers.

For the apple—*N. ditissima* pathosystem, very little is known about the pathogenicity factors of the pathogen or the resistance mechanisms of the host. Recent work using the cultivar “Royal Gala” has demonstrated that there are strains of *N. ditissima* that are almost non-pathogenic and others that are pathogenic, though it is not yet known whether the nearly non-pathogenic isolates are more pathogenic on other cultivars (Scheper et al., 2015). It is also unknown how resistance may be expressed in different tissues of the host, e.g., wood vs. fruit. It may be that resistance mechanisms are localized at the leaf scar, an area that is vulnerable to pathogen attack, as many reports have shown variation in susceptibility of leaf scar infections (Alston, 1970; Amponsah et al., 2015).

*Malus s*pecies and apple cultivars show variation in susceptibility to *N. ditissima* (Alston, 1970; Van De Weg, 1987; van de Weg, 1989; Ghasemkhani et al., 2015) though most modern varieties are susceptible. Variations in disease susceptibility may partly be a result of disease escape, e.g., the speed of wound healing in relation to *N. ditissima* infection has been shown to differ between cultivars (Xu et al., 1998). Other studies have shown that variation in colonization rate is important for resistance responses (Van De Weg, 1987; van de Weg, 1989). There is clear evidence in some breeding material of a genetic based resistance with resistance controlled predominantly by additive gene action (Gelvonauskiene et al., 2007). This offers the potential to map quantitative trait loci (QTL) in progeny segregating for disease resistance (i.e., derived from parents which have high resistance and susceptibility). Once discovered, QTLs may be cloned and the underlying resistance mechanism determined through functional genomics. Ultimately, molecular markers designed close to the QTLs, or in the causal resistance genes can be utilized in breeding programmes. At Plant and Food Research (PFR), evaluation of a germplasm sub-set showed that “Robusta 5,” “Golden Delicious,” “Priscilla,” and “Close” have good levels of resistance and are good candidates for future QTL studies (Bus et al., in press).

No specific molecular resistance mechanisms have yet been reported to *N. ditissima*. It is therefore unknown whether basal defenses are consitutively higher in resistant cultivars, or whether the strength or breadth of downstream induced resistance responses contributes to quantitative variation in resistance to *N. ditissima*. Variation in loci implicated in basal resistance, for example allelic varition in clusters of germin-like proteins, have been implicated in quantitative resistance in other systems, indicating that both the complement of basal defense genes and the strength of the induced responses are important (Manosalva et al., 2009). It is important to understand not only the genetic architecture of resistance (and the tissues in which it is expressed), but also the mechanism by which the pathogen is detected by the host. In a classical gene-for-gene system, loss or mutation of genes or motifs within proteins in the pathogen, that the plant uses to recognize and activate defenses (*R*-gene mediated resistance), results in a loss of resistance (Jones and Dangl, 2006). This model applies equally to major gene resistance or a quantitative gene-for-gene model. Work done with *Phytophthora infestans* late-blight and the cultivated potato, *Solanum tuberosum* is a current example of quantitative gene-for-gene resistance which is dependent upon recognition of multiple RxLR-containing pathogen effector genes (Rietman et al., 2012). Similar examples of quantitative *R*-gene mediated resistance have been reported in *Oryza sativa*-*Magnaporthe* interactions (Liu et al., 2011) and non-host resistance in pepper against *P. infestans* (Lee et al., 2014). It is likely that *R* genes may also underpin resistance to *N. ditissima*, coupled to a MAMP-triggered immune response, i.e., basal defense below the level required to activate the hypersensitve response (HR) as reported for the SCFE1/RLP30 interaction with the necrotrophic pathogen *Sclerotinia sclerotiorum* and *Arabodpsis thaliana* (Zhang et al., 2013). Alternatively resistance could also follow an inverse gene-for-gene model (Fenton et al., 2009), whereby resistance genes act as factors that the pathogen may exploit to activate HR deliberately, as in the case of *Botrytis cinerea* and other necrotrophic pathogens, in order to provide a nutrient source for the pathogen (Govrin and Levine, 2000). In this case, loss of recognition by *R* genes would lead to lack of HR and therefore a loss of susceptibility. Either of these is a possiblity and is one of the fundamental questions that remains to be addressed in this pathosystem. In order to assess the durability of resistance (Vleeshouwers et al., 2011) it is important to understand what evolutionary constraints pathogen genes that a host may recognize are under. By assessing the likelihood that these genes can be lost, i.e., whether they are dispensable or indispensable for pathogen virulence (polymorphic for presence/absence in a pathogenic population), under relaxed selective constraints, or able to rapidly adapt (elevated non-synonymous polymorphisms or substitutions), an assessment can be made about whether these factors are likely to rapidly evolve to evade recognition.

The objective of this study was to identify patterns of nucleotide diversity in a global sample of *N. ditissima* to understand whether there are significant differences between geographically distinct populations and to develop methods to study pathogen variability and host responses using a range of inoculation tests with apple material at different developmental stages. Understanding the patterns of nucleotide diversity of different populations of *N. ditissima* along with key population genetic parameters, such as the level of recombination within populations, gene flow between populations and the likely origin of different subpopulations is key when considering how to deploy resistance in a globally grown commodity crop such as apple. Due to the lack of regionally adapted ideotypes in apple, it is likely that the same cultivar could be grown in all areas of the world. It is therefore important to understand the level of pathogen variability and whether there are significant differences between the levels of standing genetic variation, sexual reproduction (and hence efficacy of selection) and pathogenicity of locally prevalent pathogenic isolates.

## Materials and methods

### Locus identification for phylogenetic analysis

Existing primer sets from Marra and Corwin (2009), Shivas and Tan (2009), Gräfenhan et al. (2011) and Armitage et al. (2015) were BLASTed to the R09/05 genome sequence in order to identify loci (Gómez-Cortecero et al., 2015). Extracted regions were then BLASTed to the N305S21 and N324S12 *N. ditissima* genomes (Deng et al., 2015). Hits to the three genomes were examined for polymorphism and primers that contained no polymorphic sites between isolates with an amplicon length of approximately 500 bp were designed. Primers were BLASTed back to the R09/05 genome to ensure they only hit a single gene region. In short, primers to a CDP-diacyglycerol-3-phosphate-phosphatidyltransferase (CDP) (Armitage et al., 2015) spanning the second and third exons were designed, along with primers to an intergenic region upstream of NdCAA4, named NDCAA4_prox (Marra and Corwin, 2009), primers within the same gene in which NdCAA11 primers mapped (spanning the first intron, named NDCAA11_sub) and primers from a putative ATP-citrate synthase subunit 2 gene which multi-species ACL1 primers hit (again spanning the first intron; Shivas and Tan, 2009; Gräfenhan et al., 2011). Primer sequences are shown in Supplementary Table 1.

### DNA extraction and PCR amplification

*N. ditissima* mycelium was grown in YPD liquid media (20 g Bacto peptone, 10 g yeast extract, 950 mL of water, 50 mL of 40% w/v glucose). A sterile toothpick was used to scrape young mycelia of *N. ditissima* from an agar plate and to inoculate a flask with 20 ml of YPD. The flask was closed with a cotton gauze and covered with aluminum foil. The culture flask was incubated in a shaker at a constant 20°C at 120 rpm for 1 week. Cultures were then centrifuged at 5000 g and the supernatant removed. The mycelium was washed with 10 ml of sterile water and the supernatant removed after centrifugation. Liquid nitrogen was used to freeze the mycelium and 100 mg of wet weight was homogenized using ball bearings and a tissue lyser for 2 min at 15 Hz. For DNA extraction the Macherey-Nagel NucleoSpin Plant II kit was used following a modified manufacturer's protocol.

The PCR reaction mixture contained 1 μl of gDNA (5 ng/μl), 0.5 μM of each primer, 0.625U of Taq polymerase, 0.2 mM of dNTPs, 1X PCR buffer and water to a final volume of 25 μl. The thermal profile used for the amplifications was slightly different depending upon the primers used. For the polymorphic microsatellite loci primer pairs NDCAA4_prox and NDCAA11_sub the following amplification programme was used: 95°C for 2 min followed by 30 cycles of 95°C for 30 s and then 55°C for 1 min and 72°C for 1 min. The ACL1 and the CDP loci were amplified following the thermal profile: 95°C for 2 min followed by 25 cycles of 95°C for 30 s and then 62°C for 1 min and 72°C for 30 s.

The resulting PCR products were purified using the Macherey-Nagel NucleoSpin Gel and PCR clean-up kit following the manufacturer's protocol.

### Alignment and population analysis

Sequenced ABI reads were imported into Geneious 9.0.4 software (www.geneious.com) and forward and reverse reads were aligned and consensus called. Each gene was aligned individually using the MAFFT alignment tool within Geneious (Katoh et al., 2002) and end regions trimmed so that all isolates had complete sequence information. Alignments were exported in nexus file format and diversity statistics (π, ${\hat{\theta }}_{W}$, Tajima's *D* and the 4 gamete test) calculated in DNASP v5 (Librado and Rozas, 2009). Construction of combined SNP and microsatellite haplotypes was carried out manually.

### Inoculum preparation

Inoculum of *N. ditissima* used for all pathogenicity experiments was obtained from single ascospore cultures. Three isolates were used in the pathogenicity experiments; R09/05, Hg199, and R28/15 (Table 1).

**Table 1:** **Isolate name, origin and contribution**.

**Isolate accession**	**SYN**	**CV**	**Origin**	**Year of isolation**	**Contributor**
R09/05	–	Cox	Kent, UK	2005	Angela Berrie, EMR, UK
HG199	–	Gala	Kent, UK	1999	Angela Berrie, EMR, UK
HG23	–	Gala	Kent, UK	1999	Angela Berrie, EMR, UK
HG187/B	–	Gala	Kent, UK	1999	Angela Berrie, EMR, UK
TL109	–	Cox	Kent, UK	1999	Angela Berrie, EMR, UK
TL88	–	Gala	Kent. UK	1999	Angela Berrie, EMR, UK
M46/A	–	Various	Kent, UK	1990's	Angela Berrie, EMR, UK
R28/15	–	Gala	Hampshire, UK	2015	Angela Berrie, EMR, UK
R36/15	PCF171	Jonagold	Belgium	2006	Tom Smets, PCF, B
R37/15	PCF191	Jonagold	Belgium	1999	Tom Smets, PCF, B
R38/15	PCF188	Golden Delicious	Belgium	2006	Tom Smets, PCF, B
R40/15	–	Kanzi	The Netherlands	2015	Marcel Wenneker, WUR, NL
R41/15	–	Wellant	The Netherlands	2015	Marcel Wenneker, WUR, NL
R42/15	–	Elstar	The Netherlands	2015	Marcel Wenneker, WUR, NL
R43/15	–	Junami	The Netherlands	2015	Marcel Wenneker, WUR, NL
R44/15	–	Rubens	The Netherlands	2015	Marcel Wenneker, WUR, NL
R45/15	–	Elstar	The Netherlands	2015	Marcel Wenneker, WUR, NL
R46/15	–	Jonagold	The Netherlands	2015	Marcel Wenneker, WUR, NL
R47/15	–	Delcorf	The Netherlands	2015	Marcel Wenneker, WUR, NL
R48/15	–	Natyra	The Netherlands	2015	Marcel Wenneker, WUR, NL
NB8/15	–	Royal Gala	Santa Catarina, Brazil	2015	Hugo Medeiros, EPAGRI, BR
NB9/15	–	Royal Gala	Santa Catarina, Brazil	2015	Hugo Medeiros, EPAGRI, BR
LDPL01	RS324p	Golden Delicious	Taranaki, New Zealand	2009	Reiny Scheper, PFR, NZ
LDPK01	RS305p	Brookfield Gala	Lower Moutere, New Zealand	2009	Reiny Scheper, PFR, NZ

The isolates were sub-cultured onto SNAY media (1 g potassium dihydrogen phosphate, 1 g potassium nitrate, 0.5 g magnesium sulfate, 0.5 g potassium chloride, 0.2 g glucose, 0.2 g sucrose, 1 g yeast extract, 20 g agar made up to 1 liter with distilled water). Plates were incubated in 16/8 h light/dark regime at 22°C for 13–15 days. On the day of inoculation, each plate was flooded with 3 ml of sterile water and conidia were released from sporodochia using a plastic spreader. Mixed spore (macro and microconidia) suspension was prepared from each isolate.

Macroconidia and microconidia in the suspension were counted using a haemocytometer. Two isolates, R09/05 and Hg199, were used for the cultivar cut-shoot test with concentrations of 5 × 10^4^ and 3 × 10^3^ conidia ml^−1^, respectively. When this test was repeated, three isolates, R28/15, Hg199 and R45/15 were used at a concentration of 1 × 10^5^ conidia ml^−1^. Isolate R28/15 was used in the rootstock potted tree test at 1.1 × 10^5^ conidia ml^−1^. For the apple seedling test, isolate R09/05 was used at 2.7 × 10^5^ conidia ml^−1^. For the leaf scar inoculation test, isolate R09/05 was used at 6 × 10^5^ conidia ml^−1^.

### Cultivar cut shoot test

Shoots were inoculated with two *N. ditissima* isolates, Hg199 and R09/05 along with a water control. Dormant 1-year old shoots with a length of approximately 5 cm were collected from mature trees of “Aroma,” “Beauty of Bath,” “Cox's Orange Pippin,” “Gala,” “Gloster 69,” “Golden Delicious,” “Grenadier,” “Idared,” “M9,” “Robusta 5,” and “Wolf River” at the beginning of February 2015 (in the UK). Shoots were wrapped in moist paper and kept at 4°C in darkness for 12 weeks. Four days before inoculation, shoots were placed into a controlled environment cabinet on a 20/4 h light/dark cycle with a corresponding day/night temperature of 22/18°C at a constant humidity of 80% relative humidity (RH). Shoots were immobilized at their base in Oasis floral foam, which was placed into a tray containing water, adapted from van de Weg (1989). Three axillary buds on each shoot were inoculated. Buds were prepared by cutting just below the bud, a little below the second abscission layer (but without removing the bud). The width of the incision was approximately 2–3 mm. The chosen buds were the sixth, eighth, and tenth counting basipetally (from the apex to the base of the shoot). An inoculum volume of 10 μl of spore suspension was applied to the wound within 5 min of making the wound. Following inoculation, wounds were covered with white petroleum jelly, which was removed after 4 days with a paper towel. This step is necessary to ensure that the wound does not dry out during early establishment of infection. During the first 4 days after inoculation RH was increased to 100%, again to ensure that sufficient humidity was maintained for successful infection. The experiment was divided between two growth cabinets, within which were trays containing cut shoots of cultivars, inoculated at three points (pseudo-replicates) with one of two *N. ditissima* isolates or a water control (not included in this analysis). Within each tray a single replicate of the experiment was randomly arranged in a 6 × 2 grid (eleven cultivars were analyzed in this test); there were six biological replicates per treatment. Trays of inoculated and control material were randomized between cabinets. Lesion length was recorded using digital calipers at 12, 16, 22, 27, 31, and 35 days post-inoculation. The Area Under Disease Progress Curve (AUDPC) was calculated using the agricolae package (de Mendiburu, 2015), using R version 3.2.2 (Team, 2015). AUDPC values were analyzed using a linear mixed effects model. The fixed effects followed a three-way factorial treatment structure, which was isolate × cultivar × pseudo-rep. The random effect model was cultivar, nested within trays within growth cabinets. The REML command was used within Genstat (VSN International). Wald tests were carried out in order to assess the effect of the different fixed effects and any higher order interactions that may have occurred.

This experiment was repeated in January 2016, using a subset of cultivars inoculated with isolates R28/15, Hg199, and R45/15. The protocol differed slightly since instead of three inoculations per shoot, a single inoculation was carried out to allow lesion expansion in highly susceptible cultivars to be accurately recorded at the later stages of the experiment (14, 18, 21, 27, 34, 39, 45, 49, and 54 days post-inoculation). The data are presented from day 34, to facilitate comparison with the 2015 experiment.

### Apple seedling test

Apple seeds from biparental crosses (see Results) were washed in a weak (2%) bleach solution, sown by family in trays, with 45 seeds per tray in standard horticultural compost (peat based) and stratified for 12 weeks at 2°C. Trays were then moved to a warm glasshouse 25/16°C (day/night temperature) and 16/8 h day/night length (achieved using supplementary lighting). Seedlings were grown for 6 months under these conditions and then potted into two liter pots and moved to a chilled glasshouse in early UK summer (July) 2015, at a maximum day temperature of 20°C with no additional lighting. Misting lines were hung under benches (with 360° misting units at approximately 60 cm intervals along the underside of the bench). These were placed on a timer, spraying for 10 min at 6 h intervals to ensure a minimum humidity level of 80% RH.

Three leaves from each plant were removed; either the fifth, seventh, and ninth (or fourth, sixth, and eighth) leaves depending upon the size of the plant. The corresponding axillary bud was also removed. Inoculation points were prepared by cutting just below the bud wound, a little below the second abscission layer; the width of the incision was approximately 2–3 mm. Within 5 min of cutting, 3 μl of a conidial suspension of a single *N. ditissima* isolate was placed onto the wound with an automatic micropipette. The order of inoculation was randomized into eight different sets of seedlings for logistical reasons and eight different inoculum tubes were used, prepared from a common source. This was done to avoid prolonged use of a single tube of inoculum, or to confound position in the glasshouse of the seedlings with inoculation time. Inoculated wounds were covered with white petroleum jelly within 5 min of the droplet being absorbed which was removed 7 days later with a tissue. Lesion size was recorded with digital calipers every 3 days after the first signs of infection, in this case 11 days after infection. In total seven assessments were carried out, up to 31 days post-inoculation. Seedlings were fully randomized and divided into sets of 88 and placed on two benches either side of the glasshouse. A subset of 16 seedlings (all genetically non-identical) from eleven bi-parental crosses (total 176 seedlings) were used in this test.

### Rootstock potted tree test

The experiment was carried out in a single glasshouse compartment within which were fifteen randomized blocks of five rootstock types (all 2-year old trees) with temperature, light and humidity conditions identical to the apple seedling test. Rootstocks were inoculated at three points (pseudo-replicates) with a single *N. ditissima* isolate, however this time at nodes 5, 10, and 15 to allow room for lesion expansion; this allowed the experiment to be run for much longer than the cut shoot or seedling scion experiments. Lesion length was measured at 25, 34, 49, 74, and 96 days post-inoculation. As before, AUDPC values were analyzed using a linear mixed effects model. The fixed effects followed a two-way factorial treatment structure, which was cultivar × pseudo-rep. The random effect model was cultivar, nested within blocks. REML analysis was carried out as described.

### Leaf scar inoculation potted tree test

Dormant 1-year-old shoots from mature trees of “Aroma,” “Golden Delicious,” “Gala,” “Gloster 69,” “Grenadier,” “Robusta 5,” “M9,” “E93-79,” “E202-6,” and “Idared” were grafted onto M9 rootstocks in February 2015 (UK). Trees were moved to a glasshouse 1 day before inoculation, at the end of October 2015. Temperature varied in the glasshouse from 10°C to 25°C and no additional lights were used during the experiment. To ensure a minimum humidity level of 80% RH, misting lines were hung over the trees spraying for 30 min at 6 h intervals. On each tree, five leaves were removed randomly along the tree leaving approximately the same distance among them. An inoculum volume of 10 μl of spore suspension or water control was applied to each leaf scar. The position of the trees in the glasshouse and the order of inoculation was randomized in five different sets with one tree per cultivar, inoculating four sets with a single *N. ditissima* isolate and one with water. After 5 weeks, trees were moved outside keeping the same randomized design. The first symptoms of infection appeared 70 days post-inoculation and lesion length was recorded using digital calipers at approximately fortnightly intervals. The experiment was ended at 115 days post-inoculation.

## Results

### Population analysis of *N. ditissima* reveals only slight evidence for geographically structured populations

Little is known about the extent or patterns of nucleotide diversity of *N. ditissima*, or whether there are any patterns of isolation by distance on a local or a global scale. In order to study this isolates of *N ditissima* gathered from the UK, Netherlands, Belgium, New Zealand, and Brazil were evaluated at four single copy loci found to be polymorphic in the three recently published *N. ditissima* reference genomes (Deng et al., 2015; Gómez-Cortecero et al., 2015). These loci span the introns of two conserved genes (ACL1 and CDP) and two microsatellite-containing loci, one (CAA4_prox) within an intergenic region and another (CAA11_sub) within a hypothetical protein-encoding region (Supplementary Table 1). The latter two loci were developed based on the earlier work of Marra and Corwin (2009). For each locus, between 20 and 22 isolates were evaluated originating from the UK (8 isolate), Belgium (3 isolates) the Netherlands (9 isolates), Brazil (2 isolates), and New Zealand (2 isolates) (Table 1).

The number of segregating SNP sites varied between 2 and 12 and estimates of π, a measure of nucleotide diversity, ranged by approximately an order of magnitude (0.002–0.018), depending upon the locus (Table 2). In all but one case, Tajima's *D* (a comparison of the scaled mean number of pairwise differences and the number of segregating sites) revealed no evidence for selective or demographic processes acting on the chosen loci. However, in the case of the CDP gene, where a clear haplotype containing 11/12 SNPs can be seen, there is a significantly positive measure of Tajima's D, indicative of non-neutral patterns of nucleotide polymorphism.

**Table 2:** **Population genetics statistics for a global sample of ***N. ditissima*****.

**Gene**	**Samples**	**Sites**	**Sites for SNP**	**Segregating**	**π (Average pairwise differences per site)**	**$\hat{\theta }$*W* (Watterson's theta- segregating sites)**	**Tajima's D (SNP)**	**Haplotypes (SNP)**	**Private SNP (origins)**	**Haplotypes incl Microsat**	**Private SNP and microsatellite haplotypes (origins)**	**Evidence for recombination**
ACL1	21	409	409	4	0.00447	0.00272	1.84 (ns)	3	Belgium	NA	Belgium	No
CDP	22	339	339	12	0.01794	0.00971	2.97 (*p* < 0.01)	3		NA		No
NDCAA4_prox	22	321	297	2	0.00155	0.00185	−0.37 (ns)	3		7	Brazil, Netherlands, New Zealand, UK	No
NDCAA11_sub	20	362	278	9	0.00841	0.00913	−027 (ns)	3	Netherlands	7	Brazil, UK and Netherlands	No

The number of SNP haplotypes was the same (3) in each locus under study and only two private SNP haplotypes were found (SNPs found only in a single subpopulation), one in the Netherlands, in a single individual, for the CAA11_sub locus and one in a Belgian isolate for the ACL1 locus, indicating that most polymorphism is shared between populations (Table 2). Including both SNP and microsatellite variation (for which the mutation rate per cell division may be over twice as high 7 × 10^−8^) in the analysis of private haplotypes reveals that despite the small sample size, distinct private haplotypes could also be detected in UK, Netherlands, Brazilian, and New Zealand samples (Table 2, Supplementary Table 2). Across all samples no evidence for recombination within loci could be detected using the four-gamete test (Hudson and Kaplan, 1985), however segregation could be detected between loci.

### Differences in partial resistance to canker among cut shoots of apple cultivars

It is widely known that cultivars vary in their susceptibility to canker, though the exact molecular mechanism is unknown. In order to further study the response of cultivars to different inocula, different infection methods and at different physiological conditions, a pathogenicity screen using two UK isolates (R09/05 and Hg199) was carried out first, using dormant cut shoot material (van de Weg, 1989). This test allows colonization rate to be calculated and compared between isolates and cultivars. After inoculation, lesions progressed vertically along the shoots. The symptoms consisted of a sunken and necrotic bark area around the inoculation point, the progress of which was measured in a non-destructive manner with calipers. These symptoms were noticeable after 12 days after inoculation in the cut-shoot test. Using REML analysis followed by tests for fixed effects, no effect of the growth cabinet could be seen (the experimental design explicitly controlled for this eventuality). Cut shoot tests revealed abundant variation in resistance and susceptibility to *N. ditissima*, but little variation in isolate pathogenicity (Table 3). This variation in the response among the cultivars was consistent regardless of the differences in the inoculation pressure between the isolates (see also Supplementary Figure 1). There was a significant effect of pseudo-replicate position (three inoculation points per scion were used).

**Table 3:** **Wald tests for fixed effects- sequentially adding terms to fixed model**.

**Fixed effect**	**Wald statistic**	**d.F**.	**Chi pR**
Cabinet	0.01	1	0.91
Isolate	0.9	1	0.34
Cultivar	108.24	11	**2 × 10^−16^**
Pseudo-replicate	6.50	2	**0.04**
Isolate:Cultivar	16.72	11	0.12
Isolate:Pseudo-replicate	0.67	2	0.71
Cultivar:Pseudo-replicate	17.64	22	0.73
Isolate:Cultivar:Pseudo-replicate	28.30	22	0.17

*Significant differences are highlighted in bold*.

**Figure 2:**
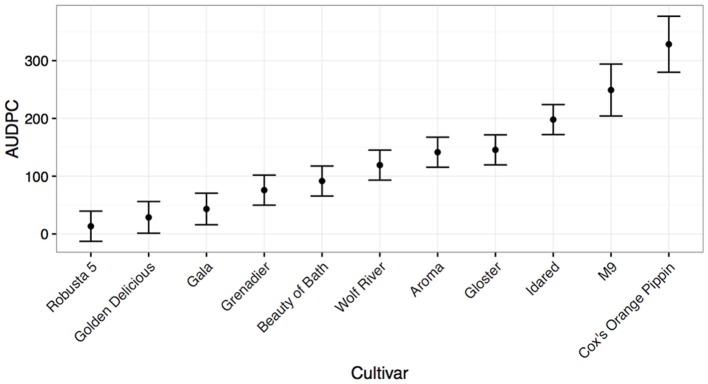
**Mean Area under disease progress for inoculated cut shoots of common apple scion material calculated 35 days post-infection (shown with standard errors)**. The rootstock M9 is also included as a qualitative comparison.

For apple scion material, it was found that the species *Malus* × *robusta* c.v. “Robusta 5” had the highest level of resistance in the cut shoot tests (Figure 2), followed by the known resistant cultivar “Golden Delicious.” At the other end of the resistance spectrum, the known susceptible cultivars “EMLA-'M9” (a rootstock) and “Cox” were highly susceptible (Figure 2). Intermediate levels of resistance were seen for other reported field-resistant or tolerant material, including “Aroma,” “Beauty of Bath,” and “Grenadier.” Somewhat surprisingly the field-susceptible cultivar, “Gala” was found to be more resistant than expected to *N. ditissima* infection using this method. Based on its reported parentage (“Golden Delicious” × “Kidd's Orange Red”- the latter reported to be a “Delicious” × “Cox” cross), it has both resistant and presumed susceptible material in its pedigree indicating the potential for at least partial resistance, consistent with the performance of “Golden Delicious” and “Cox” in this test). Repetition of this experiment in 2016 with three isolates of *N. ditissima* revealed similar results, with “Gala” and its offspring “Scifresh” and “Scilate” (“Gala” × “Braeburn”) all showing low levels of lesion spread (Supplementary Figure 1) and no cultivar by isolate interaction (data not shown).

### Differences in partial resistance to canker among apple rootstocks

**Figure 3:**
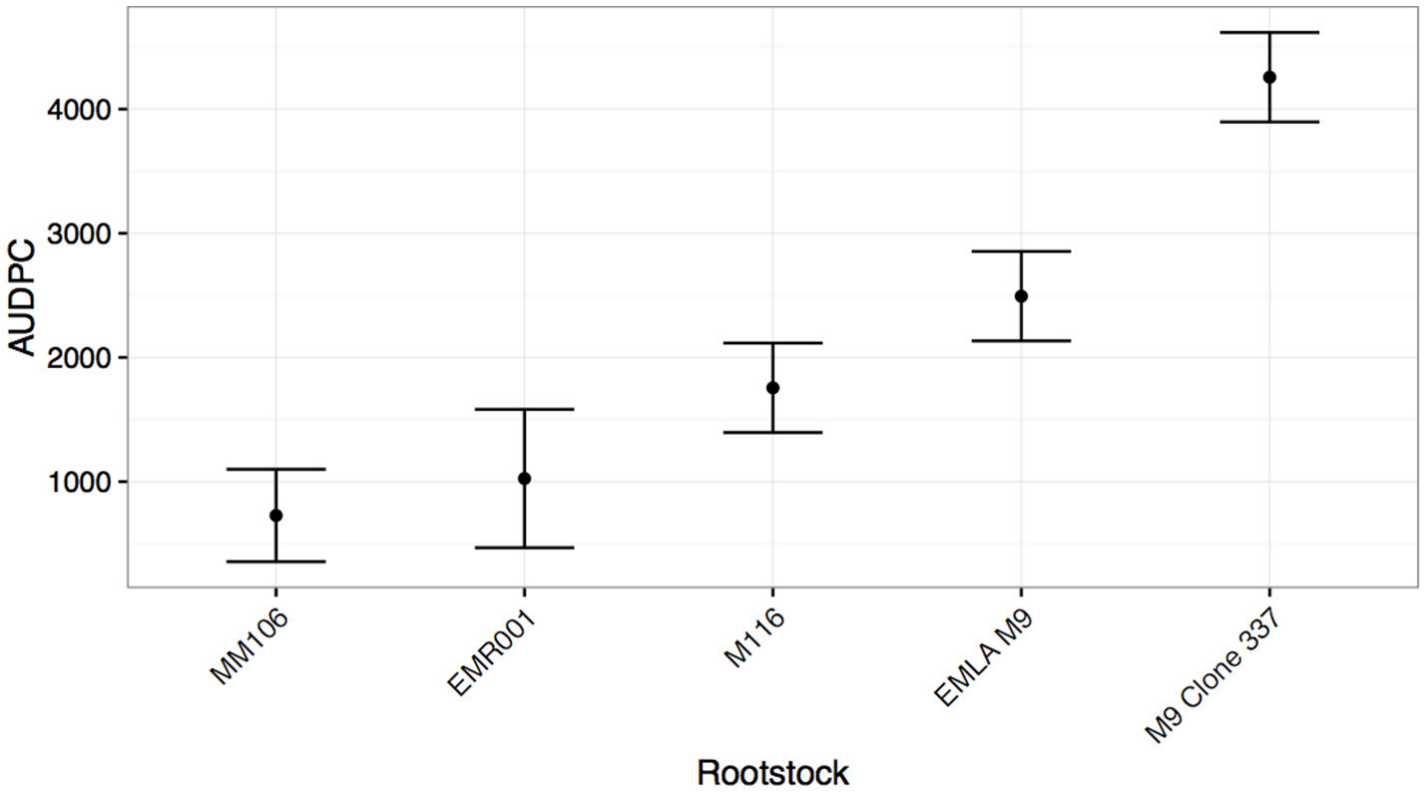
**Mean Area under disease progress for inoculated shoots of common apple rootstocks (shown with standard errors), calculated 75 days post-infection**.

As with the cultivar test, significant effects of both rootstock cultivar and pseudo-replicate were detected, though this time a significant two-way interaction between cultivar and pseudo-replicate was detected (Table 4). In this experiment, five rootstocks were tested (including two clonal variants of “M9”). “MM106” (“M2” × “Northern Spy”) was the most resistant, while the “M9” clone (337) was the most susceptible (Figure 3).

**Table 4:** **Wald tests for fixed effects- sequentially adding terms to fixed model**.

**Fixed effect**	**Wald statistic**	**d.F**.	**Chi pR**
Cultivar	64.82	4	**2.8 × 10^−13^**
Pseudo-replicate	52.57	2	**3.5 × 10^−12^**
Cultivar.Pseudo-replicate	25.24	8	**0.0014**

*Significant differences are highlighted in bold*.

### Differences in partial resistance to canker determined by leaf scar inoculation

**Figure 4:**
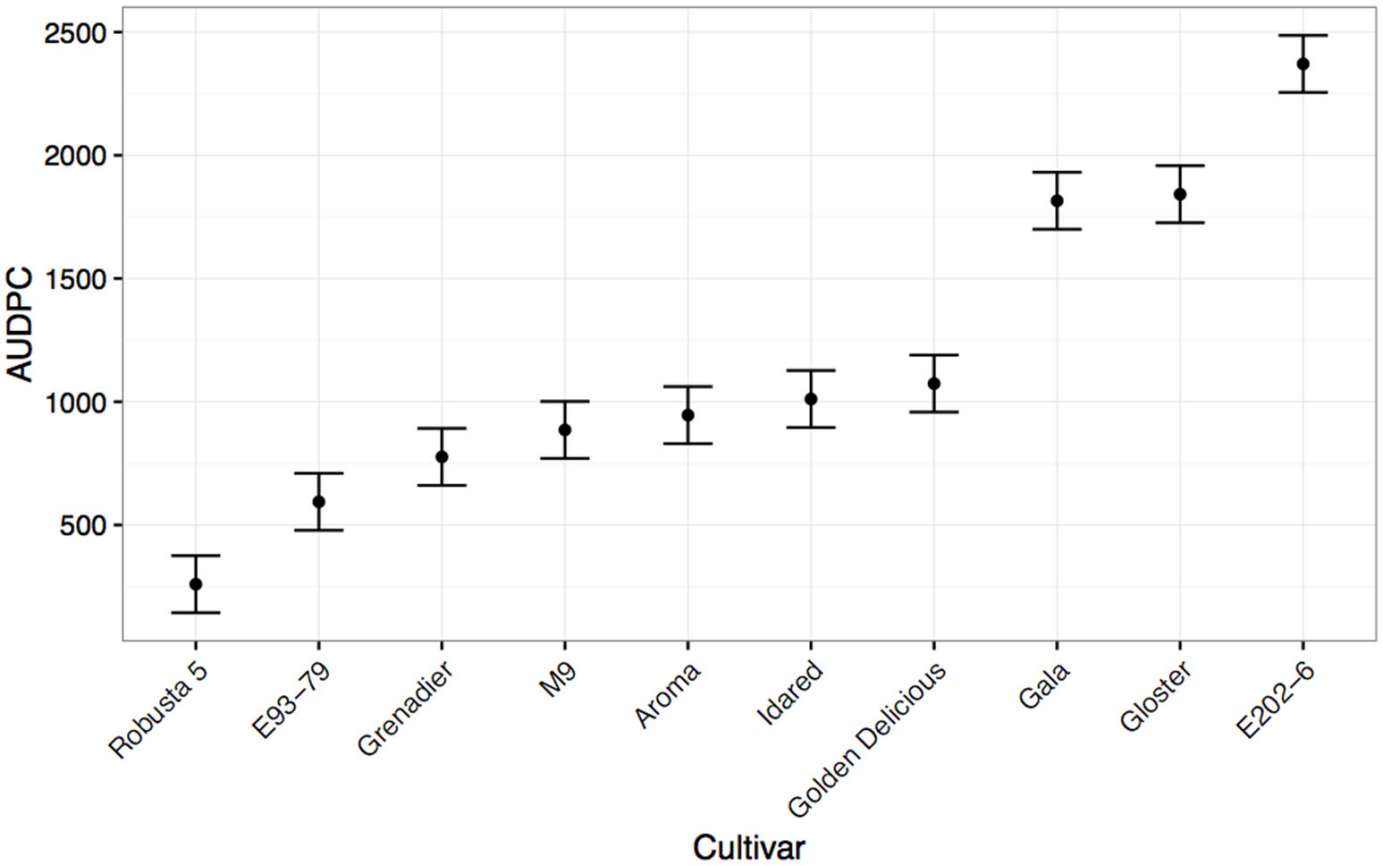
**Mean Area under disease progress for inoculated leaf scars of common apple scion material calculated 153 days post-infection (shown with standard errors)**. The rootstock M9 is also included as a qualitative comparison.

Alongside cut shoot tests, leaf scar infection tests were carried out (Alston, 1970; Amponsah et al., 2015; Scheper et al., 2015). Again, the species level accession “Robusta 5” demonstrated high levels of resistance (Figure 4). As with previous reports, “Gala” was extremely susceptible in this pathogenicity test, with high levels of colonization after inoculation with the same isolate of *N. ditissima* as used in the cut shoot test (Scheper et al., 2010). “Gloster 69” and “E202-6” also showed high levels of susceptibility. Intermediate levels of resistance were seen in “Golden Delicious,” “Idared,” “Aroma,” “M9,” “Grenadier,” and “E93-79.”

### Seedling tests indicate a complex genetic basis for resistance

**Figure 5:**
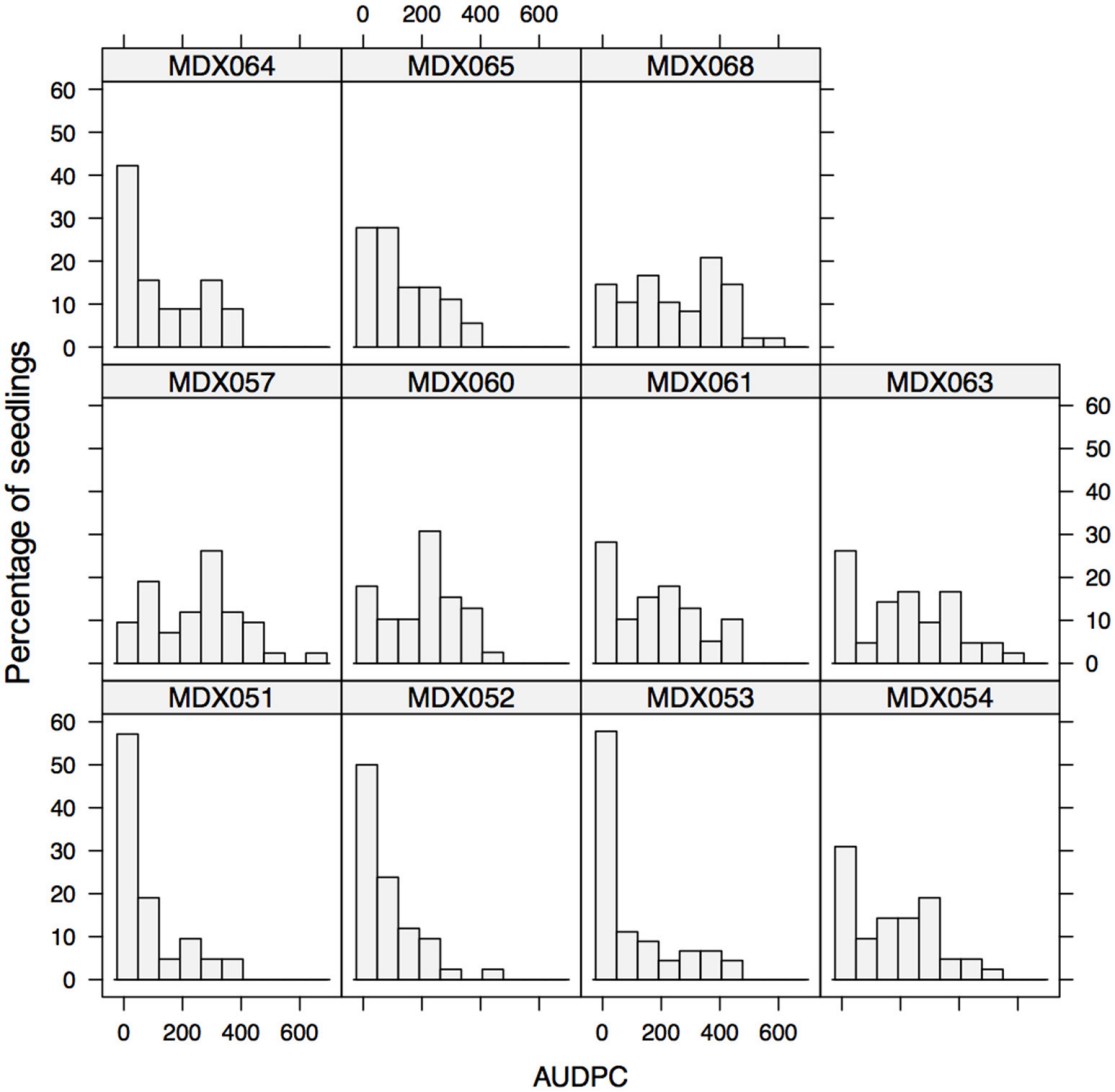
**Histogram of AUDPC values for inoculated shoots of eleven seedling families of ***M. x domestica*****. Segregation patterns vary considerably between families and unexpected segregation patterns occur between crosses of highly resistant varieties.

In order to further test the resistance responses of different parental material with respect to variation in colonization rate following wound inoculation, and the manner in which resistance is transmitted, crosses were made between parents, many of which were tested in a cut shoot test. The experiment was run for a total of 31 days; significant symptom development was seen in some progenies 11 days after inoculation. Examination of the AUDPC values after 31 days revealed that segregation patterns varied and crosses with both highly resistant offspring (MDX053 and MDX051 having the lowest median AUDPC values) and highly susceptible offspring (MDX057, MDX068) were observed (Figure 5 and Table 5).

**Table 5:** **Cross combinations tested in the seedling test and population median and inter-quartile range of the AUDPC values**.

**Cross number**	**Female parent**	**Male parent**	**Population median (AUDPC)**	**Population IQR (AUDPC)**	**Cross combination resistance based on cut-shoot R- resistant, I- intermediate, S- susceptible, U- unknown (F × M)**
MDX051	Gala	Santana	28.58	101.5	R × U
MDX052	Aroma	Gala	60.41	108.8	I × S
MDX053	Aroma	Fuji	25.92	148.41	I × U
MDX054	Aroma	Golden Delicious	181.42	248.95	I × R
MDX057	Gloster 69	Idared	266.61	227.03	I × S
MDX060	E248-2	E616-57	234.06	185.85	U × U
MDX061	E93-79	Gala	180.53	251.23	U × S
MDX063	E202-6	Golden Delicious	244.59	292.96	U × S
MDX064	Gala	3760	85.8	242.69	U × R
MDX065	Gala	3762	105.05	158.53	S × U
MDX068	Grenadier	Golden Delicious	253.39	251.96	I × R

The segregation patterns that were observed were complex and some resistant parents showed poor transmission of resistance into the progeny. For example, crosses involving “Golden Delicious,” even when crossed with other moderately resistant parental lines (e.g., “Aroma,” MDX054 and “Grenadier,” MDX068) showed higher median levels of disease progress (Median AUDPC 181.42) than crosses involving the same parental material (e.g., Aroma) crossed to more susceptible material (e.g., “Gala,” MDX052- Median AUDPC 60.41), though significant differences were only observed in a single pairwise non-parametric Kruskal Wallis test between MDX052 and MDX068, but not MDX052 × MDX054 and MDX054 × MDX068 (see Supplementary Table 3).

The four most resistant crosses involved “Gala,” “Santana,” “Aroma,” “Fuji,” and “3760”- the latter an open pollinated line derived from *M*. × *robusta* (see Supplementary Table 4 for pedigree details).

## Discussion

Our data, although incomplete, present a pattern of SNP diversity consistent with the notion that there is broad similarity between geographically isolated populations and that much of the genetic diversity seen in the European population of *N. ditissima* is also seen in South American and Oceanian populations. At the SNP level (with an approximate mutation rate of the order of 3 × 10^−8^ for nuclear base substitutions per cell division, Lynch et al., 2008) there is little information about recent geographic isolation, as there is no clear pattern of private allelic variation with geographic origin. These data are consistent with the idea that *N. ditissima* spread from Europe to other regions of the world on imported apple plant material. Further study will be needed with much larger sample sizes to provide estimates of local levels of diversity in these populations. Despite the small sample size, it was possible to detect with the aid of more rapidly evolving microsatellite loci evidence for some distinct patterns of polymorphism in UK, Netherlands, Brazilian, and New Zealand populations of *N. ditissima* (Table 2 and Supplementary Table 2). Again, further sampling will be needed to confirm this, but again the pattern of differentiation at microsatellites is suggestive that local populations of *N. ditissima* with unique allelic variation may be detectable with more polymorphic loci.

Interestingly, despite *N. ditissima* being a sexually reproducing species, with a year-round reproductive potential no evidence for recombination within loci could be detected in our sequenced isolates, though segregation between loci can be seen. Other recent work has proven that recombination does occur in the field by SSR tests of ascospores (Ghasemkhani et al., 2016). Further data are needed to study the genome-wide patterns of recombination to determine whether the patterns observed from the loci used in this study are general to the whole genome. The levels of nucleotide diversity vary widely and there are signatures of non-neutral processes (evidenced by significantly positive Tajima's D values). A key question to address in the future is whether these patterns are present across the whole genome; if so this would be indicative of demographic processes influencing patterns of nucleotide diversity. In the model system *Saccharomyces cerevisiae* it was shown that “modern” wine and baking yeast strains were admixed individuals with contributions from multiple different subpopulations, previously allopatrically isolated (Liti et al., 2009). This pattern was postulated to be driven by human influences, bringing geographically isolated strains together by human migration. It is conceivable that *N. ditissima* has also experienced similar human-driven secondary contact in the recent evolutionary past, which has contributed to the extant patterns of nucleotide diversity. In order to test this hypothesis, multiple samples from across Europe, the Americas and Oceania, as well as other areas of the world where *N. ditissima* is established must be sequenced and subjected to population genomics analysis. Alternatively, it could be that different populations of *N. ditissima* have expanded their host-ranges onto apple to create hybrid recombining populations. It is important to undertake population-level analyses as genome-wide association studies of pathogenicity may be confounded by high levels of ancestral population structure.

The finding from both preliminary cultivar cut shoot tests, that there is no isolate by cultivar interaction, suggests that the host response is consistent, regardless of the isolate that is inoculated. To confirm these initial observations, a further study of more isolates is required. All higher order interactions were non-significant, indicating that there may be a relatively simple pattern of host response which is not influenced by an isolate race-structure, consistent with previous reports (van de Weg, 1989). The finding that there was a positional effect of pseudo-replicate can be explained by the fact that in some cultivars lesion growth was so rapid, that after a point it became impossible to distinguish between lesion leading edges, at which point the experiment was ended.

While our results do not support the existence of distinct pathogen races, this has no bearing on whether the resistance that has been identified may be durable or not (as this is primarily determined by the capability of the pathogen to overcome specific defense or recognition mechanisms). However, it may suggest that resistance is targeting conserved factors in the pathogen and therefore the resistance present in the tested cultivars may be broad spectrum and thus has the potential to be durable. It is interesting to note that the most resistant cultivar “Robusta 5” is a representative of a species that is distinct from *M*. × *domestica*. Little is known about natural *M*. × *robusta* species, since much of the material that is present in Europe was collected in Northern China. It is described as a hybrid species, though this is only by morphology (Forsline et al., 2002). What is interesting to note is that *N. ditissima* is not reported as a significant pathogen of apple in China, indicating that *M*. × *robusta* may be a non-host and therefore that the mode of resistance in *M*. × *robusta* vs. the cultivated apple *M*. × *domestica* may be of distinct evolutionary origin. It is therefore important to study multiple origins of resistance, as some may be more durable than others, or pyramiding combinations of different alleles may offer greater resistance by combining multiple mechanisms of resistance.

The finding that rootstock material also has resistance to *N. ditissima* is encouraging. It has been shown that during nursery propagation, infected rootstock material may be one of the primary mechanisms by which the disease is spread and therefore rootstock material with high levels of resistance may reduce the subsequent emergence of latent *N. ditissima* in the orchard (McCracken et al., 2003). The surprising finding that M9 clones exhibit differing levels of susceptibility merits further investigation. The mechanism for this is unknown, but it could be that during the selection for clonal variants with improved propagation or yield characteristics other somatic mutations that alter the resistance response may have been inadvertently selected. This may also explain differences in field susceptibility of supposed identical cultivars. Many of the commercially grown “Gala” clones are in fact sports selected for skin color or ripening date. It may also be a risk, when considering clonally propagated crops, that resistance gene pyramids may be disrupted by somatic mutations in clonal material, which could lead to loss of resistance durability. In order to identify whether clonal propagation and selection of material leads to differences in susceptibility a more comprehensive study must be undertaken, evaluating clones produced under the same propagation conditions.

The seedling test that was carried out revealed that resistance sources differed in their transmission characteristics. Most striking was the observation that crosses involving “Golden Delicious,” found to be highly resistant in cut shoot tests, had a greater level of susceptibility when crossed to resistant material, than supposed resistant × intermediate/susceptible crosses. This could be explained if the nature of the resistance sources differed among cultivars, i.e., if the resistance from “Golden Delicious” was recessive, or if susceptibility factors in some cross combinations lead to resistance that is non-additive. These preliminary results suggest that the likelihood of transmission of resistance varies between resistant parental material and that some parental material appears to be superior to others in ability to donate resistance, despite slightly lower overall resistance in the cut shoot tests (i.e., moderately resistant “Aroma” vs. highly resistant “Golden Delicious”). This part of the study highlights the importance of trial evaluation of seedling populations prior to embarking upon QTL studies and the importance of considering the mode and mechanism of resistance and the way in which it is phenotyped in breeding programmes.

It is still unclear whether the methods that have been tested in this paper are of direct relevance to the orchard situation. The methods used in this study have not been compared with orchard inoculations and therefore it is entirely possible that a newly developed cultivar that is resistant according to these tests turns out to be susceptible in the field. It is clear that both resistance to colonization and initial infection are important components of field resistance. Some cultivars that we have studied, such as “Gala” appear to have consistently high levels of resistance to colonization in cut shoot tests (Figure 2, Supplementary Figure 1) and yet are often considered to be field susceptible and indeed in whole-tree leaf scar tests (Figure 4) are much more susceptible. Conversely, some material (see E93-79, Figure 4 and Supplementary Figure 1) exhibits rapid colonization in wound inoculated cut shoot tests, but low susceptibility to leaf scar infection. It should be noted that in both types of pathogenicity test “Robusta 5” displays low levels of infection and subsequent colonization. This suggests that the cut shoot and leaf scar tests are querying different components of resistance and that for strong resistance, low levels of colonization and lesion expansion in both tests are required. In order to be considered to be field resistant, trees must have low disease incidence when several wound types are inoculated; a small lesion size when infection does occur; low spore production from lesions; negligible internal (latent) growth of the pathogen. Future work needs to be carried out to compare the results presented in this study with trees grown outside in an orchard setting, inoculated using several different wounds (leaf scars, pruning cuts, picking wounds) to determine whether the methods developed in this paper can be considered to be sufficient for rapid selection in breeding programmes.

It is also important to consider the role of abiotic stresses in modulating plant resistance. It is unclear at present, when issues with drainage in the orchard occur or other changes in tree health, or nitrogen applications, whether the resistance status of some trees may alter more than others. It is rare that multifactorial experiments are carried out on a field scale that address biotic stress responses in relation to abiotic stress tolerance. However, a study by Dryden et al. (2016), as well as anecdotal evidence suggest that this is an important topic of future study (Dryden et al., 2016).

With the recent publication of three *N. ditissima* genome sequences (Deng et al., 2015; Gómez-Cortecero et al., 2015) and the increasing amount of genomic information available for apple (Velasco et al., 2010; Antanaviciute et al., 2012; Bianco et al., 2014; Bink et al., 2014) it is likely that rapid progress can be made in identifying the genetic basis of resistance to *N. ditissima* from multiple resistance sources and the corresponding pathogenicity factors that may be manipulating host defenses.

## Author contributions

RH conceived the study and led the writing of the manuscript, AG, JK, and RJS carried out experimental work, HA, RWAS, and JB contributed to the phylogenetic analysis. AG, RH, and XX analyzed the pathogenicity test data. All authors contributed to writing and editing the manuscript.

### Conflict of interest statement

The authors declare that the research was conducted in the absence of any commercial or financial relationships that could be construed as a potential conflict of interest.

## Supplementary material

Supplementary Figure 1 **Mean Area under disease progress for inoculated cut shoots of common apple scion material calculated 34 days post-inoculation (shown with standard errors)**. The rootstock M9 is also included as a qualitative comparison.

Supplementary Table 1 **Primer sequences used for population analysis of ***N. ditissima*****.

Supplementary Table 2 **Combined SSR and SNP haplotypes reported by population of origin**.

Supplementary Table 3 **Multiple comparison of seedling populations using Kruskal-Wallis tests**.

Supplementary Table 4 **Pedigree relationships between seedling material evaluated for canker resistance**. Color coding indicates generation- black (F1), red (F0), blue (F-1) orange (F-3), green (F-4), and purple (F-5) generations. Inter-generational crosses are highlighted by the color of the generation in which they first appear.

## References

[B1] Alston F. (1970). Response of apple cultivars to canker, *Nectria galligena*. Annu. Rep. East Malling Res. Stn. 1969 A53, 147–148.

[B2] Amponsah N. T. Walter M. Beresford R. M. Scheper R. W. A. (2015). Seasonal wound presence and susceptibility to *Neonectria ditissima* infection in New Zealand apple trees. N.Z. Plant Prot. 68, 250–256.

[B3] Antanaviciute L. Fernández Fernández F. Jansen J. Banchi E. Evans K. M. Viola R.. (2012). Development of a dense SNP-based linkage map of an apple rootstock progeny using the *Malus* Infinium whole genome genotyping array. BMC Genomics 13:203. 10.1186/1471-2164-13-203 22631220 PMC3410780

[B4] Armitage A. D. Barbara D. J. Harrison R. J. Lane C. R. Sreenivasaprasad S. Woodhall J. W.. (2015). Discrete lineages within Alternaria alternata species group: identification using new highly variable loci and support from morphological characters. Fungal Biol. 119, 994–1006. 10.1016/j.funbio.2015.06.012 26466875

[B5] Bianco L. Cestaro A. Sargent D. J. Banchi E. Derdak S. Di Guardo M.. (2014). Development and validation of a 20K single nucleotide polymorphism (SNP) whole genome genotyping array for apple (*Malus* × *domestica* Borkh). PLoS ONE 9:e110377. 10.1371/journal.pone.0110377 25303088 PMC4193858

[B6] Bink M. C. A. M. Jansen J. Madduri M. Voorrips R. E. Durel C.-E. Kouassi A. B.. (2014). Bayesian QTL analyses using pedigreed families of an outcrossing species, with application to fruit firmness in apple. Theor. Appl. Genet. 127, 1073–1090. 10.1007/s00122-014-2281-3 24567047

[B7] Bus V. Singla G. Ward S. Brewer L. Morgan C. Bowatte D. (in press). Progress in pipfruit resistance breeding research at Plant & Food Research. Acta Hortic. 27427289

[B8] Castlebury L. A. Rossman A. Y. Hyten A. S. (2006). Phylogenetic relationships of *Neonectria/Cylindrocarpon* on *Fagus* in North America. Can. J. Bot. 84, 1417–1433. 10.1139/b06-105

[B9] Cayley D. M. (1921). Some observations on the life-history of *Nectria galligena*, Bres. Ann. Bot. 35, 79–92.

[B10] Cooke L. R. (1999). The influence of fungicide sprays on infection of apple cv. Bramley's seedling by *Nectria galligena*. Eur. J. Plant Pathol. 105, 783–790. 10.1023/A:1008778900607

[B11] de Mendiburu F. (2015). agricolae: Statistical Procedures for Agricultural Research. R Package Version 1.2-3. Available online at: http://CRAN.R-project.org/package=agricolae

[B12] Deng C. H. Scheper R. W. A. Thrimawithana A. H. Bowen J. K. (2015). Draft genome sequences of two isolates of the plant-pathogenic fungus *Neonectria ditissima* that differ in virulence. Genome Announc. 3:e01348–15. 10.1128/genomeA.01348-15 26586888 PMC4653790

[B13] Dryden G. H. Nelson M. A. Smith J. T. Walter M. (2016). Postharvest foliar nitrogen applications increase *Neonectria ditissima* leaf scar infection in apple trees. N.Z. Plant Prot. 69, 230–237.

[B14] Fenton A. Antonovics J. Brockhurst M. A. (2009). Inverse-gene-for-gene infection genetics and coevolutionary dynamics. Am. Nat. 174, E230–E242. 10.1086/645087 19852618

[B15] Forsline P. L. Aldwinckle H. S. Dickson E. E. Luby J. J. Hokanson S. C. (2002). Collection, maintenance, characterization, and utilization of wild apples of Central Asia, in Horticultural Reviews: Wild Apple and Fruit Trees of Central Asia, Vol. 29, ed Janick J. (Oxford, UK: John Wiley & Sons, Inc.), 1–62. 10.1002/9780470650868.ch1

[B16] Gelvonauskiene D. Sasnauskas A. Gelvonauskis B. Gelvonauskiene D. Sasnauskas A. Gelvonauskis B. (2007). The breeding of apple tree resistant to European Canker (*Nectria galligena* Bres). Sci. Work. Lith. Insitute Hortic. Lith. Univ. Agric. 26, 174–178.

[B17] Ghasemkhani M. Garkava-Gustavsson L. Liljeroth E. Nybom H. Bernier L. Hubbes M. (2016). Assessment of diversity and genetic relationships of *Neonectria ditissima*: the causal agent of fruit tree canker. Hereditas 153, 7 10.1186/s41065-016-0011-3 PMC5226109 28096769

[B18] Ghasemkhani M. Liljeroth E. Sehic J. Zborowska A. Nybom H. (2015). Cut-off shoots method for estimation of partial resistance in apple cultivars to fruit tree canker caused by *Neonectria ditissima*. Acta Agric. Scand. Sect. B Soil Plant Sci. 65, 412–421. 10.1080/09064710.2015.1016101

[B19] Gómez-Cortecero A. Harrison R. J. Armitage A. D. (2015). Draft genome sequence of a European isolate of the apple canker pathogen *Neonectria ditissima*. Genome Announc. 3, 10–11. 10.1128/genomeA.01243-15 26586869 PMC4653771

[B20] Govrin E. M. Levine A. (2000). The hypersensitive response facilitates plant infection by the necrotrophic pathogen *Botrytis cinerea*. Curr. Biol. 10, 751–757. 10.1016/S0960-9822(00)00560-1 10898976

[B21] Gräfenhan T. Schroers H.-J. Nirenberg H. I. Seifert K. A. (2011). An overview of the taxonomy, phylogeny, and typification of nectriaceous fungi in Cosmospora, Acremonium, Fusarium, Stilbella, and Volutella. Stud. Mycol. 68, 79–113. 10.3114/sim.2011.68.04 21523190 PMC3065986

[B22] Hudson R. R. Kaplan N. L. (1985). Statistical properties of the number of recombination events in the history of a sample of DNA sequences. Genetics 111, 147–164. 4029609 10.1093/genetics/111.1.147 PMC1202594

[B23] Jones J. D. G. Dangl J. L. (2006). The plant immune system. Nature 444, 323–329. 10.1038/nature05286 17108957

[B24] Katoh K. Misawa K. Kuma K. Miyata T. (2002). MAFFT: a novel method for rapid multiple sequence alignment based on fast Fourier transform. Nucleic Acids Res. 30, 3059–3066. 10.1093/nar/gkf436 12136088 PMC135756

[B25] Lee H.-A. Kim S.-Y. Oh S.-K. Yeom S.-I. Kim S.-B. Kim M.-S.. (2014). Multiple recognition of RXLR effectors is associated with nonhost resistance of pepper against *Phytophthora infestans*. New Phytol. 203, 926–938. 10.1111/nph.12861 24889686 PMC4143959

[B26] Librado P. Rozas J. (2009). DnaSP v5: a software for comprehensive analysis of DNA polymorphism data. Bioinformatics 25, 1451–1452. 10.1093/bioinformatics/btp187 19346325

[B27] Liti G. Carter D. M. Moses A. M. Warringer J. Parts L. James S. A.. (2009). Population genomics of domestic and wild yeasts. Nature 458, 337–341. 10.1038/nature07743 19212322 PMC2659681

[B28] Liu Y. Zhu X. Y. Zhang S. Bernardo M. Edwards J. Galbraith D. W.. (2011). Dissecting quantitative resistance against blast disease using heterogeneous inbred family lines in rice. Theor. Appl. Genet. 122, 341–353. 10.1007/s00122-010-1450-2 20872132

[B29] Lynch M. Sung W. Morris K. Coffey N. Landry C. R. Dopman E. B.. (2008). A genome-wide view of the spectrum of spontaneous mutations in yeast. Proc. Natl. Acad. Sci. U.S.A. 105, 9272–9277. 10.1073/pnas.0803466105 18583475 PMC2453693

[B30] Manosalva P. M. Davidson R. M. Liu B. Zhu X. Hulbert S. H. Leung H.. (2009). A germin-like protein gene family functions as a complex quantitative trait locus conferring broad-spectrum disease resistance in rice. Plant Physiol. 149, 286–296. 10.1104/pp.108.128348 19011003 PMC2613727

[B31] Marra R. E. Corwin J. A. (2009). Isolation and characterization of codominant markers for the perennial canker fungal pathogen *Neonectria ditissima*. Mol. Ecol. Resour. 9, 906–909. 10.1111/j.1755-0998.2008.02438.x 21564786

[B32] McCracken A. R. Berrie A. Barbara D. J. Locke T. Cooke L. R. Phelps K. (2003). Relative significance of nursery infections and orchard inoculum in the development and spread of apple canker (*Nectria galligena*) in young orchards. Plant Pathol. 52, 553–566. 10.1046/j.1365-3059.2003.00924.x

[B33] Plante F. Hamelin R. C. Bernier L. (2002). A comparative study of genetic diversity of populations of *Nectria galligena* and *N. coccinea* var. faginata in North America. Mycol. Res. 106, 183–193. 10.1017/S0953756201005329

[B34] Rietman H. Bijsterbosch G. Cano L. M. Lee H.-R. Vossen J. H. Jacobsen E.. (2012). Qualitative and quantitative late blight resistance in the potato cultivar Sarpo Mira is determined by the perception of five distinct RXLR effectors. Mol. Plant Microbe Interact. 25, 910–919. 10.1094/MPMI-01-12-0010-R 22414442

[B35] Scheper R. W. A. Fisher B. M. Wood P. N. (2010). Pathogenicity of field and laboratory-grown inoculum of *Neonectria galligena* on potted apple trees. N.Z. Plant Protect. 63:280.

[B36] Scheper R. W. A. Frijters L. Fisher B. M. Hedderley D. I. (2015). Effect of freezing of *Neonectria ditissima* inoculum on its pathogenicity. N.Z. Plant Prot. 68, 257–263.

[B37] Shivas R. Tan Y. (2009). A taxonomic re-assessment of Colletotrichum acutatum, introducing *C. fioriniae* comb. et stat. nov. and *C. simmondsii* sp. nov. Fungal Divers. 39, 111–112.

[B38] Swinburne T. R. (1975). European canker of apple (*Nectria galligena*). Rev. Plant Pathol. 54, 787–799.

[B39] Team R. C. (2015). R: A Language and Environment for Statistical Computing. Vienna: R Foundation for Statistical Computing Available online at: https://www.R-project.org/

[B40] Tulasne L. Tulasne C. (1865). Selecta Fungorum Carpologica III Nectriei-Phacidiei-Pezizei. Paris: Imperial: English Translation by WB Grove 1931; Oxford: Clerendon.

[B41] Van De Weg W. E. (1987). Note on an inoculation method to infect young apple seedlings with *Nectria galligena* Bres. Euphytica 36, 853–854. 10.1007/BF00051869

[B42] van de Weg W. E. (1989). Screening for resistance to *Nectria galligena* Bres. in cut shoots of apple. Euphytica 42, 233–240. 10.1007/BF00034459

[B43] Velasco R. Zharkikh A. Affourtit J. Dhingra A. Cestaro A. Kalyanaraman A.. (2010). The genome of the domesticated apple (*Malus* × *domestica* Borkh.). Nat. Genet. 42, 833–839. 10.1038/ng.654 20802477

[B44] Vleeshouwers V. G. A. A. Raffaele S. Vossen J. H. Champouret N. Oliva R. Segretin M. E.. (2011). Understanding and exploiting late blight resistance in the age of effectors. Annu. Rev. Phytopathol. 49, 507–531. 10.1146/annurev-phyto-072910-095326 21663437

[B45] Walter M. Glaister M. K. Clarke N. R. Von Lutz H. Eld Z. Amponsah N. T. (2015). Are shelter belts potential inoculum sources for *Neonectria ditissima* apple tree infections? N.Z. Plant Prot. 68, 227–240.

[B46] Weber R. W. S. (2014). Biology and control of the apple canker fungus *Neonectria ditissima* (syn. *N. galligena*) from a Northwestern European perspective. Erwerbs Obstbau 56, 95–107. 10.1007/s10341-014-0210-x

[B47] Xu X. Butt D. J. Ridout M. S. (1998). The effects of inoculum dose, duration of wet period, temperature and wound age on infection by *Nectria galligena* of pruning wounds on apple. Eur. J. Plant Pathol. 104, 511–519.

[B48] Xu X.-M. Robinson J. D. (2010). Effects of fruit maturity and wetness on the infection of apple fruit by *Neonectria galligena*. Plant Pathol. 59, 542–547. 10.1111/j.1365-3059.2009.02232.x

[B49] Zhang W. Fraiture M. Kolb D. Löffelhardt B. Desaki Y. Freddy F. G.. (2013). Arabidopsis RECEPTOR-LIKE PROTEIN30 and receptor-like kinase SUPPRESSOR OF BIR1-1/EVERSHED mediate innate immunity to necrotrophic fungi. Plant Cell 25, 4227–4241. 10.1105/tpc.113.117010 24104566 PMC3877809

