# Supplementary figures and images for: Variation in Host and Pathogen in the Neonectria/Malus Interaction; toward an Understanding of the Genetic Basis of Resistance to European Canker
Source: Front Plant Sci. 2016 Sep 15;7:1365. doi: 10.3389/fpls.2016.01365 (PMC5023678; doi:10.3389/fpls.2016.01365)

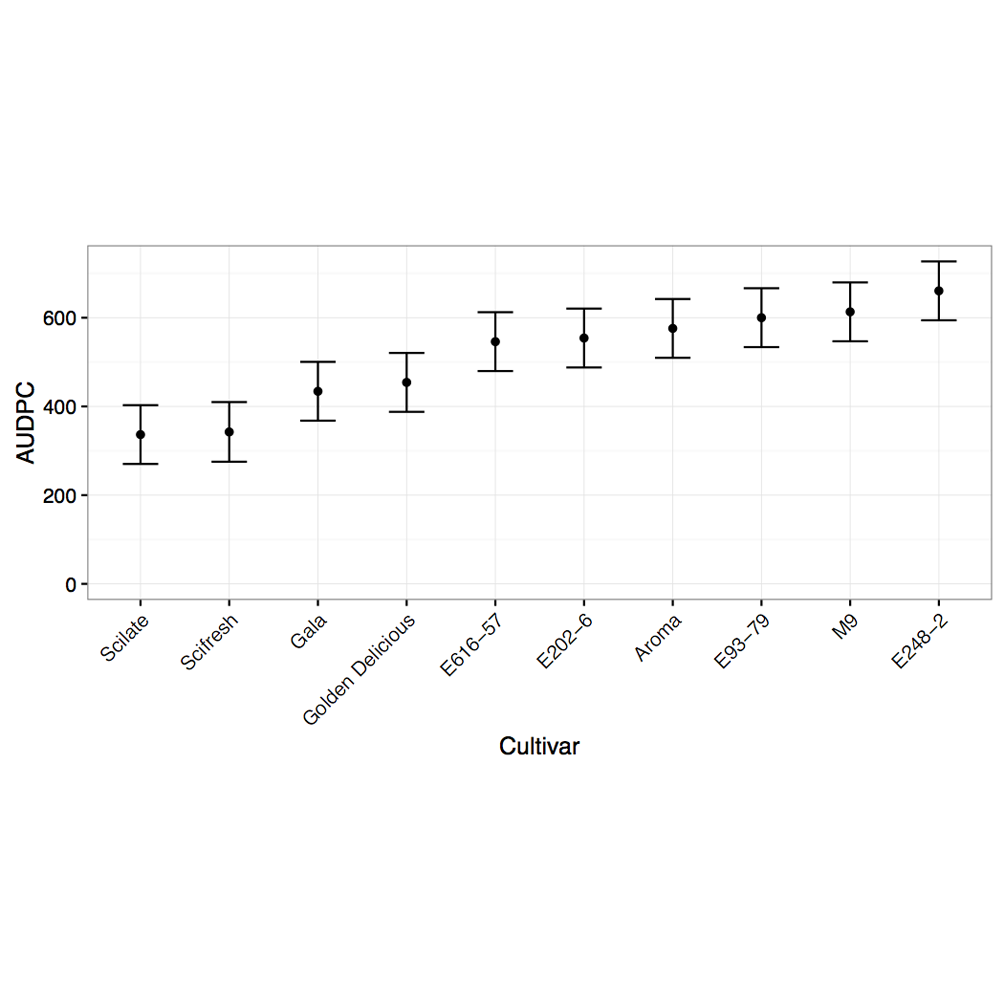

Supplement: Supplementary Figure 1 — Mean Area under disease progress for inoculated cut shoots of common apple scion material calculated 34 days post-inoculation (shown with standard errors). The rootstock M9 is also included as a qualitative comparison. [file Image1.TIFF]
