# Supplementary material for: Variation in Host and Pathogen in the Neonectria/Malus Interaction; toward an Understanding of the Genetic Basis of Resistance to European Canker
Source: Front Plant Sci. 2016 Sep 15;7:1365. doi: 10.3389/fpls.2016.01365 (PMC5023678; doi:10.3389/fpls.2016.01365)
Supplement: Supplementary Table 1 — Primer sequences used for population analysis of N. ditissima. [file Table1.DOCX]

Supplementary Table 1: Primer sequences used for population analysis of *N. ditissima*

| **Locus^a.^** | **Primer** | **Primer sequence (5'-3')** | **Primer source** | **Annealing** | **Amplicon length** |
| --- | --- | --- | --- | --- | --- |
|  |  |  |  | **temperature (°C)** | **(approx. no. bp)** |
| *ACL1* |  |  |  | 60C | 563bp |
|  | *ACL1_sub_F* | CTCACCCTCAAGACACCGAG | This study |  |  |
|  | *ACL1_sub_R* | GGGTACTCAGCGAGGTCAAC | This study |  |  |
| *CDP* |  |  |  | 62.7C | 493bp |
|  | *CDP_F* | CAACACCCCGGGCCTCAG | This study |  |  |
|  | *CDP_R* | CGAGTTGGACTGGGGCCA | This study |  |  |
| *NdCAA4_prox* |  |  |  | 60C | 455bp |
|  | *NdCAA4_prox_F* | TTGATCATCACCCCCTCCCT | This study |  |  |
|  | *NdCAA4_prox_R* | GCAACTTGGCAAGTGGGAAA | This study |  |  |
| *NdCAA11_sub* |  |  |  | 60C | 479bp |
|  | *NdCAA11_ext (5’-3’)* | CCTCCCGCCGATGAGAAAAT | This study |  |  |
|  | *NcCAA11_ext (5’-3’)* | TTGACCGAGGTTAGCGTTCG | This study |  |  |

1. Putative ATP-citrate synthase subunit 2 CDS (ACL1), CDP-diacylglycerol--glycerol-3-phosphate 3-phosphatidyltransferase (CDP), Intergenic region containing CAA tri-nucleotide repeat (*NdCAA4)*, Genic region containing CAA tri-nucleotide repeat (*NdCAA11_sub),*
