# Supplementary material for: Variation in Host and Pathogen in the Neonectria/Malus Interaction; toward an Understanding of the Genetic Basis of Resistance to European Canker
Source: Front Plant Sci. 2016 Sep 15;7:1365. doi: 10.3389/fpls.2016.01365 (PMC5023678; doi:10.3389/fpls.2016.01365)
Supplement: Supplementary Table 2 — Combined SSR and SNP haplotypes reported by population of origin. [file Table2.DOCX]

Supplementary Table 2: Combined SSR and SNP haplotypes reported by population of origin

| **Combined Haplotype** | **Strain** | **Country** | **Gene** |
| --- | --- | --- | --- |
| Haplotype 1 | Hg23, Hg187, R41/15 | UK, NL | **NDCAA4_prox** |
| Haplotype 2 | LDPL01, TL109, Hg199, R28/15, R36/15, R38/15 | UK,NZ |  |
| Haplotype 3 | LDPK01 | NZ |  |
| Haplotype 4 | R46/15, R48/15, M46/A, TL88, R37/15 | UK, NL |  |
| Haplotype 5 | NB8/15, NB9/15 | BR |  |
| Haplotype 6 | R09/05 | UK |  |
| Haplotype 7 | R40/15, R42/15, R44/15, R45/15 | NL |  |
| Haplotype 1 | Hg23, Hg187/B, R42/15, R48/15, TL88 | UK, NL | **CDP** |
| Haplotype 2 | Hg199, LDPK01, R09/05, R36/15, R37/15, R38/15, R40/15, R44/15, R45/15, R46/15 | UK, NZ, BE, NL |  |
| Haplotype 3 | LDPL01, M46/A, NB8/15, NB9/15, R28/15, R41/15, TL109 | UK, NZ, BR, NL |  |
| Haplotype 1 | Hg23, Hg187/B, Hg199, LDPL01, NB8/15, NB9/15, R28/15, R36/15, R38/15, R41/15, R44/15, R45/15, R46/15, TL109 | UK, BR, NZ,NL,BE | **ACL1** |
| Haplotype 2 | LDPK01, M46/A, R09/05, R42/15, R48/15', TL88 | UK,NL,NZ |  |
| Haplotype 3 | R37/15 | BE |  |
| Haplotype 1 | Hg23, Hg187/B, Hg199, LDPK01, LDPL01, R36/15, R38/15, R40/15, R41/15, R43/15, TL109 | UK, BE | **NDCAA11_sub** |
| Haplotype 2 | M46/A, R37/15 | UK, BE |  |
| Haplotype 3 | NB8/15, NB9/15 | BR |  |
| Haplotype 4 | R09/05 | UK |  |
| Haplotype 5 | R42/15, TL88 | NL |  |
| Haplotype 6 | R46/15 | NL |  |
| Haplotype 7 | R48/15 | NL |  |
