# Supplementary material for: Variation in Host and Pathogen in the Neonectria/Malus Interaction; toward an Understanding of the Genetic Basis of Resistance to European Canker
Source: Front Plant Sci. 2016 Sep 15;7:1365. doi: 10.3389/fpls.2016.01365 (PMC5023678; doi:10.3389/fpls.2016.01365)
Supplement: Supplementary Table 3 — Multiple comparison of seedling populations using Kruskal-Wallis tests. [file Table3.DOCX]

Supplementary Table 3- Multiple comparison test after Kruskal-Wallis test.

| Pairwise comparison | obs.dif | critical.dif | difference |
| --- | --- | --- | --- |
| MDX051-MDX052 | 3.27 | 96.65 | FALSE |
| MDX051-MDX053 | 9.31 | 95.02 | FALSE |
| MDX051-MDX054 | 89.37 | 96.65 | FALSE |
| MDX051-MDX057 | 144.11 | 96.65 | TRUE |
| MDX051-MDX060 | 119.00 | 98.49 | TRUE |
| MDX051-MDX061 | 91.59 | 98.49 | FALSE |
| MDX051-MDX063 | 108.83 | 96.65 | TRUE |
| MDX051-MDX064 | 44.00 | 95.02 | FALSE |
| MDX051-MDX065 | 50.37 | 100.59 | FALSE |
| MDX051-MDX068 | 139.55 | 93.58 | TRUE |
| MDX052-MDX053 | 6.04 | 95.02 | FALSE |
| MDX052-MDX054 | 86.10 | 96.65 | FALSE |
| MDX052-MDX057 | 140.83 | 96.65 | TRUE |
| MDX052-MDX060 | 115.73 | 98.49 | TRUE |
| MDX052-MDX061 | 88.32 | 98.49 | FALSE |
| MDX052-MDX063 | 105.56 | 96.65 | TRUE |
| MDX052-MDX064 | 40.72 | 95.02 | FALSE |
| MDX052-MDX065 | 47.09 | 100.59 | FALSE |
| MDX052-MDX068 | 136.28 | 93.58 | TRUE |
| MDX053-MDX054 | 80.06 | 95.02 | FALSE |
| MDX053-MDX057 | 134.80 | 95.02 | TRUE |
| MDX053-MDX060 | 109.69 | 96.89 | TRUE |
| MDX053-MDX061 | 82.28 | 96.89 | FALSE |
| MDX053-MDX063 | 99.52 | 95.02 | TRUE |
| MDX053-MDX064 | 34.69 | 93.37 | FALSE |
| MDX053-MDX065 | 41.06 | 99.03 | FALSE |
| MDX053-MDX068 | 130.24 | 91.90 | TRUE |
| MDX054-MDX057 | 54.74 | 96.65 | FALSE |
| MDX054-MDX060 | 29.63 | 98.49 | FALSE |
| MDX054-MDX061 | 2.22 | 98.49 | FALSE |
| MDX054-MDX063 | 19.46 | 96.65 | FALSE |
| MDX054-MDX064 | 45.37 | 95.02 | FALSE |
| MDX054-MDX065 | 39.00 | 100.59 | FALSE |
| MDX054-MDX068 | 50.18 | 93.58 | FALSE |
| MDX057-MDX060 | 25.11 | 98.49 | FALSE |
| MDX057-MDX061 | 52.52 | 98.49 | FALSE |
| MDX057-MDX063 | 35.27 | 96.65 | FALSE |
| MDX057-MDX064 | 100.11 | 95.02 | TRUE |
| MDX057-MDX065 | 93.74 | 100.59 | FALSE |
| MDX057-MDX068 | 4.56 | 93.58 | FALSE |
| MDX060-MDX061 | 27.41 | 100.30 | FALSE |
| MDX060-MDX063 | 10.17 | 98.49 | FALSE |
| MDX060-MDX064 | 75.00 | 96.89 | FALSE |
| MDX060-MDX065 | 68.64 | 102.36 | FALSE |
| MDX060-MDX068 | 20.55 | 95.48 | FALSE |
| MDX061-MDX063 | 17.24 | 98.49 | FALSE |
| MDX061-MDX064 | 47.59 | 96.89 | FALSE |
| MDX061-MDX065 | 41.23 | 102.36 | FALSE |
| MDX061-MDX068 | 47.96 | 95.48 | FALSE |
| MDX063-MDX064 | 64.83 | 95.02 | FALSE |
| MDX063-MDX065 | 58.47 | 100.59 | FALSE |
| MDX063-MDX068 | 30.72 | 93.58 | FALSE |
| MDX064-MDX065 | 6.37 | 99.03 | FALSE |
| MDX064-MDX068 | 95.55 | 91.90 | TRUE |
| MDX065-MDX068 | 89.18 | 97.65 | FALSE |
